# A core program of gene expression characterizes cancer metastases

**DOI:** 10.18632/oncotarget.22240

**Published:** 2017-11-02

**Authors:** Franz Hartung, Yunguan Wang, Bruce Aronow, Georg F. Weber

**Affiliations:** ^1^ University of Cincinnati Academic Health Center, Cincinnati, OH, USA; ^2^ Computational Medicine Center, Cincinnati Children's Hospital, Cincinnati, OH, USA

**Keywords:** metastasis, metabolism, vascularization, extracellular matrix, ion homeostasis

## Abstract

While aberrant expression or splicing of metastasis genes conveys to cancers the ability to break through tissue barriers and disseminate, the genetic basis for organ preference in metastasis formation has remained incompletely understood. Utilizing the gene expression profiles from 653 GEO datasets, we investigate whether the signatures by diverse cancers in various metastatic sites display common features. We corroborate the meta-analysis in a murine model. Metastases are generally characterized by a core program of gene expression that induces the oxidative metabolism, activates vascularization/tissue remodeling, silences extracellular matrix interactions, and alters ion homeostasis. This program distinguishes metastases from their originating primary tumors as well as from their target host tissues. Site-selectivity is accomplished through specific components that adjust to the target micro-environment. The same functional groups of gene expression programs are activated in the metastases of B16-F10 cells to various target organs. It remains to be investigated whether these genetic signatures precede implantation and thus determine organ preference or are shaped by the target site and are thus a consequence of implantation. Conceivably, chemotherapy of disseminated cancer might be more efficacious if selected to match the genetic makeup of the metastases rather than the organ of origin by the primary tumor.

## INTRODUCTION

The organ preference in cancer metastasis has been a subject of study and numerous hypotheses for over 125 years. In 1889, the English surgeon Stephen Paget described the propensity of various types of cancer to form metastases in specific organs, which led him to coin the metaphor that the patterns of outgrowth were due to the dependence of the “seeds” (the cancer cells) on the “congenial soil” (the target organ for metastasis) [1]. Even though the analogy continues to be cited in abundance, it has provided absolutely no mechanistic insight. Later, the pathologist James Ewing formulated a more scientific explanation by suggesting that circulatory patterns (of blood and lymph vessels) between a primary tumor and specific secondary organs were sufficient to account for most of the targeted metastases [2]. His model implied a passive role for the cancer cells that are released from their primary tumors and – consistent with their large size and low deformability – get stuck in the first capillary bed they encounter. However, serial passage of a melanoma cell line through mice generated sub-lines with increasing invasive potential and demonstrated that metastasis formation depends – at least in part – on intrinsic characteristics within the transformed cells [3]. Since the mid-1980s [4–6], it has become increasingly clear that the phenomenon of cancer metastasis can be directed by gene expression programs within the tumor cells. Metastatic potential is acquired by these tumor cells through the aberrant expression or splicing of stress response genes [7, 8]. Further, it has been elucidated that beside the positive regulators of dissemination there are gene regulation programs for metastasis suppression [9–11], which need to be inactivated for cancers to disseminate.

In regard to colorectal cancer, there is a knowledge base for the molecular characteristics of metastases to various sites. Genomic signatures are conserved in colorectal liver metastases [12] and include chromosome 20p11 gains [13]. The chemokine receptor CCL-7 [14], the adhesion molecule P-Cadherin [15], the growth factor IGF2 and the intestinal stem cell-specific transcription factor ASCL2 [12] are over-expressed, whereas MMP1 and MMP2 are under-expressed [16] in liver metastasis compared with the primary tumors. 46 genes are differentially regulated between hepatic and pulmonary metastases [17]. A gene expression signature that characterizes lung foci involves the upregulation of FN1, CCL7, MMP7, IGF1, VEGFA, and SRC [18]. The CTHRC1 (Collagen Triple Helix Repeat Containing 1) gene is associated with peritoneal carcinomatosis [19]. The expression of mesenchyme forkhead 1 (FOXC2) in the primary tumor correlates with the degree of lymph node metastasis [20].

A barrier to understanding the genetic signatures of organ preference has been the inherent heterogeneity and clonal evolution of malignant tumors. Studies of breast cancer have increasingly focused on this phenomenon [21]. While a shared pathophysiology is implied by somatic mutations within three genes (TP53, PIK3CA, GATA3) that occur in more than 10% of cases, distinct subsets of genetic and epigenetic abnormalities generate four main breast cancer types [22]. Many mutational processes emerge late, but contribute extensive genetic variation. While most genetic alterations arise in just a fraction of tumor cells, there is a dominant subclonal lineage in every tumor that represents more than half of its cells. The expansion of this dominant subclone to an appreciable mass may represent the final rate-limiting step in breast cancer progression [23]. Tumor cell dissemination, primarily into bone marrow, is an early event in the disease history [24]. There are extensive biomarker differences between the primary tumor and its metastases, as well as among multiple metastases from the same patient. Estrogen and progesterone receptors tend to be down-regulated in the metastatic growths. Variable overexpression occurs in the colonies for cyclooxygenase-2 (COX2), epidermal growth factor receptor (EGFR), MET, and mesothelin [25].

Tumor-host interactions contribute to determining organ preference in metastasis. Circulating cancer cells can recognize target organs through the use of tissue markers [26], such as the interactions of addressins or chemokines with their cognate receptors [27, 28]. The binding motifs of tri-peptides or tetra-peptides on the vasculature may also provide homing signals [29]. As such, the sequence SRL encodes a brain homing motif, CGFE is a motif in lung homing peptides recognized by membrane dipeptidase, and the integrin binding RGD motif homes to sites of neovascularization.

Despite variation from tumor heterogeneity, clonal evolution and host factors, consistent genetic adjustments are required within the tumor cells to complete the process of metastasis. Although research over the past fifteen years has identified some genes, the expression of which is associated with cancer dissemination to specific sites [30–32, 17, 18], gene regulation maps that reflect organ specificity in cancer spread are still lacking. Here we expand on earlier microarray analyses [33, 34] and investigate metastasis-specificity in gene expression signatures from various primary tumors, their metastases, and host tissue in target sites to identify genetically encoded programs that distinguish the disseminated growths.

## RESULTS

### Gene expression differences delineate metastases from their primary cancers and from their target tissues

**Figure 1:**
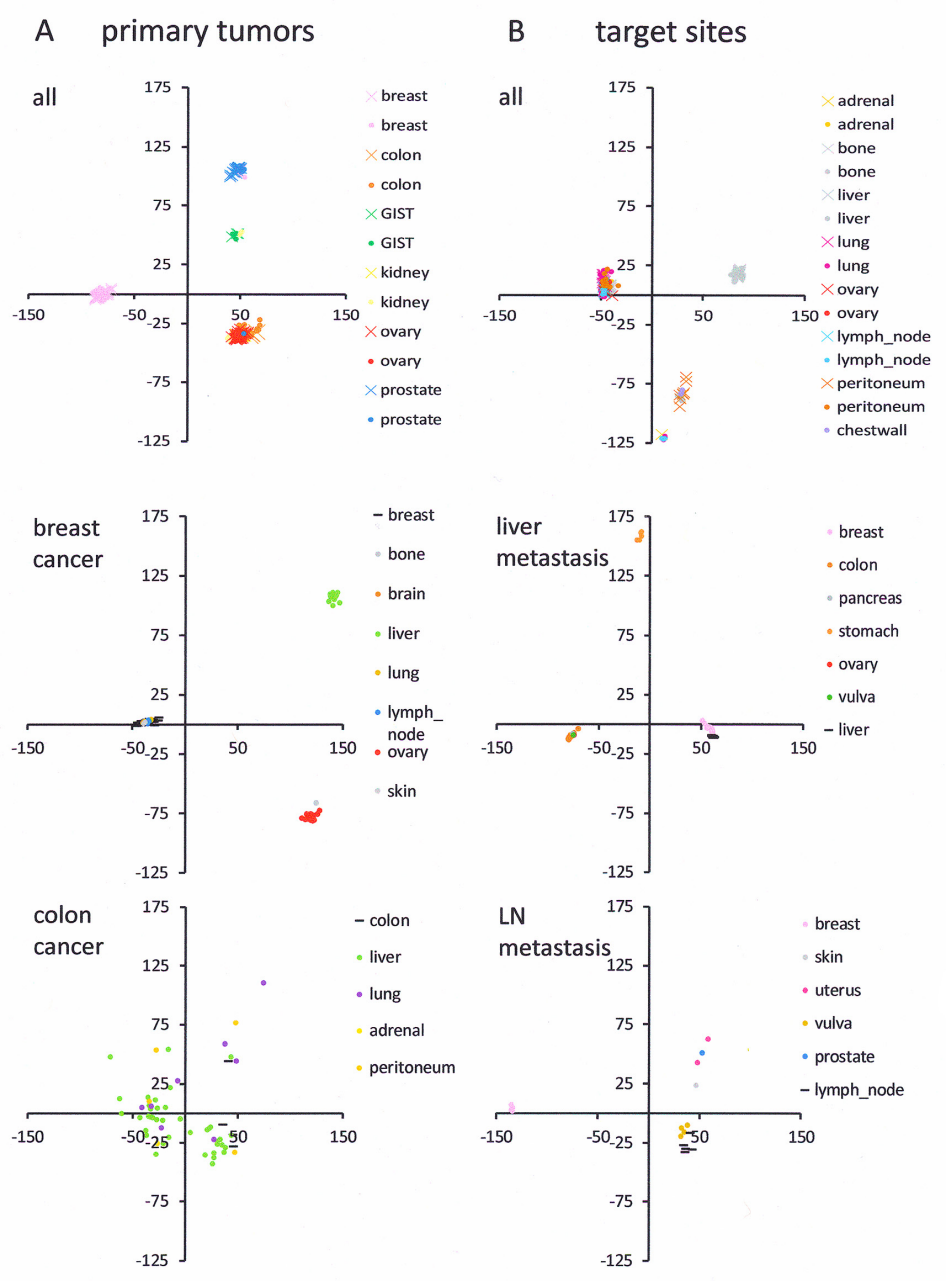
Principal component analysis **(A)** Comparison of primary tumors to their metastases in various organs. **(B)** Comparison of healthy host tissue in metastatic target sites to the metastases in those sites, having originated from various primary tumors. The top graph (A, B) shows the PCA results of the entire data set (the metastases are represented by color-filled circles, the reference values for primary tumors in A and for host tissue in B are depicted as color-matched x, while each gene expression profile is one data point there is overlap). The graphs for the individual sites of cancer origin or individual metastatic target sites (middle and bottom) are subset analyses (the PCA was done separately for each subset shown), selectively for a specific primary tumor and its colonies (A) or a specific healthy host tissue and metastases that target it (B). The primary tumor sites (A) or the host sites (B) are displayed as horizontal black lines. The round symbols, indicating the principal component coordinates for the metastases, are color coded by target site (A) or source tumor (B). Note that all graphs (x-axis PC1 and y-axis PC2) have the same scale to facilitate the comparison of cluster tightness. With the exception of colon cancer, the PC1 contribution was 50-65% and the PC2 contribution was 15-22%.

To assess overall relatedness across GEO data sets (Supplement 1), we compared samples by principal component analysis. The existence of tight clusters suggests shared features among the metastases; their frequent grouping away from primary tumors and from host tissues implies uniqueness of the metastatic gene expression (top graphs in Figures 1A and 1B). Breast cancer (primary tumor and metastases) displays three distinct clusters, comprising metastases that group far from the primary tumors and only select target sites that group with their primary tumors. Colon cancer has metastases that spread broadly away from the primary tumors (Figure 1A). While metastases to liver and lymph nodes mostly group at a distance to the host tissue (with the exception of breast cancer metastases to the liver and vulva metastases to the lymph nodes), they form distinct clusters (Figure 1B), implying the existence of common genetically encoded programs that uniquely characterize the metastases. We set out to identify the features that set metastases apart from their sources and from their targets.

### Metastases are characterized by gene expression changes in four functional groups

**Figure 2:**
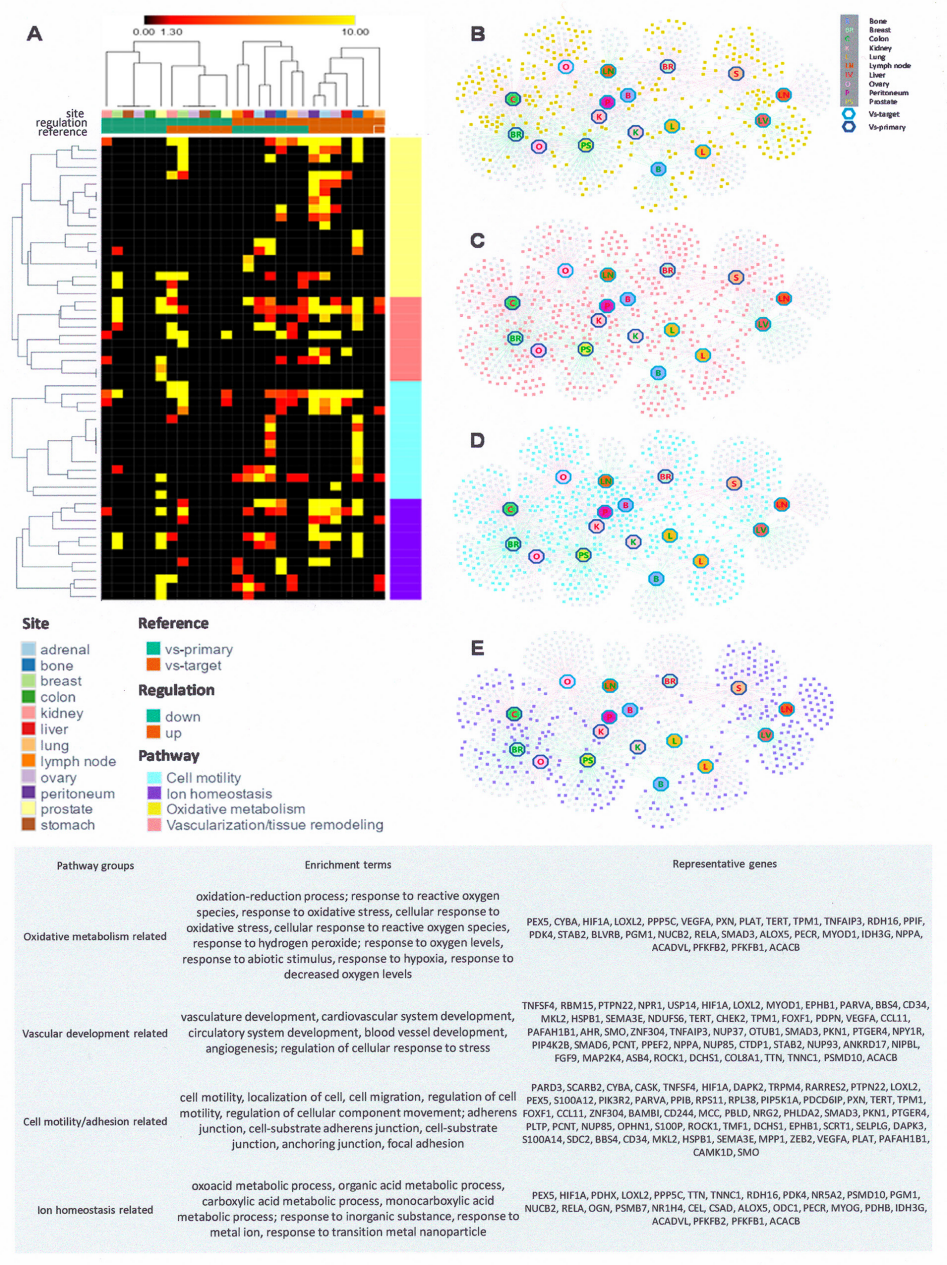
The gene expression core program of metastasis **(A)** Heatmap of enrichment terms. Differentially expressed genes in metastases (as compared to their originating primary tumors or to their target host sites) are augmented in four major pathways (colored sectioning on the right). Each row represents an enriched pathway group and each column represents a specific analysis. The columns are arranged as indicated in the bars on the top; the map has been ordered with hierarchical clustering in Spearman correlation distance. **(B-E)** Networks of differentially expressed genes that are involved the four major pathways of metastasis. Each square represents a gene, and each hexagon represents metastases that up-regulate (red label) or down-regulate (green label) those genes compared with either primary cells (dark blue border) or target cells (light turquoise border). (B) Genes involved in pathways of the oxidative metabolism are highlighted in yellow. (C) Genes involved in vascularization related pathways are highlighted in red. (D) Genes involved in pathways of cell mobility are highlighted in turquoise. (E) Genes involved in ion homeostasis related pathways are highlighted in purple. **(Bottom panel)** Table of enrichment terms (GO terms and KEGG pathways) in the four major pathway groups. Representative genes are those that are differentially expressed in at least three distinct metastasis comparisons. The functional enrichment analysis of the gene lists was performed using the ToppGene Suite.

We set up a meta-analysis type study to comprehensively evaluate relevant gene expression profiles of solid tumor metastases retrieved from GEO. The initial differential expression analysis compared metastases from various primary sites to the same target organ (all source sites combined for each target organ). Up-regulated genes identify those with higher expression levels in the metastasis samples as compared to the healthy host tissues, down-regulated genes are lower in the metastases than in the host tissue (Supplement 2). We then performed pathway enrichment analysis to identify biological processes significantly affected by the up- and down-regulated genes. Regardless of the metastatic target site, common gene expression differences from the host tissues predominantly reflect functional entities that regulate metabolism, vascularization/tissue remodeling, motility/extracellular matrix interactions, as well as pathways for inorganic ion transport/homeostasis (Figure 2). These four functional groups comprise about one fourth (25.5%) of the top changes in gene ontology categories as compared to the host organ (Supplement 2). In addition, site-specific alterations have consistent characteristics (manuscript in preparation). In sum, the gene expression profile of metastasis is characterized by a core program component plus a specific program component that adjusts to the target site.

Differential expression analysis was also performed comparing primary tumors of specific tissue origin to their respective metastases (all target sites combined for each primary tumor site). Up-regulated genes identify those with higher expression levels in the metastases as compared to the primary cancer of origin, down-regulated genes are lower in the metastases (Supplement 3). We performed pathway enrichment analysis to identify biological pathways significantly affected among up- and down-regulated genes for all metastasis sites from each source site. General changes in the metastases, compared to their primary tumors, occur predominantly in pathways that regulate metabolism, vascularization/tissue remodeling, extracellular matrix interactions/motility, and inorganic ion homeostasis (see Figure 2; Supplement 3). Further, there are changes that are unique for specific metastases-versus-primary tumor comparisons. The results were confirmed in an analysis of the largest GEO data set on breast cancer (Supplement 4).

### Metastases are distinguished from their target sites by common gene expression signatures

Select genes are up- or down- regulated compared to host tissue in all or most of the target sites analyzed (Table 1). The deregulation of numerous zinc finger proteins and other transcription factors may reflect the control by designated genetic programs. At all 7 metastatic target sites evaluated, two genes (BSN, YAF2) are induced and one gene (CKAP5) is suppressed in the metastases. Multiple genes are induced or suppressed in 5 or 6 target sites. Those sets of genes seem to constitute essential mediators of metastasis, possibly conveying the ability to survive and expand in a non-cognate microenvironment. It is implied that they are critical for determining whether a cancer forms clinically relevant metastases.

**Table 1:** Up- or down-regulated common genes in metastases compared to target

UP				DOWN			
gene symbol	target tissue	gene symbol	target tissue	gene symbol	target tissue	gene symbol	target tissue
BSN	li:pe:ov:ln:bo:ad:lu	UTP11L	pe:li:ov:bo:lu	CKAP5	li:pe:ov:ln:bo:ad:lu	FSTL4	li:ln:ov:bo:lu
YAF2	li:pe:ov:ln:bo:ad:lu	NUSAP1	li:pe:ln:ov:ad	NAB1	li:pe:ov:bo:ad:lu	CDC37L1	pe:li:ov:ln:bo
MYO9B	li:pe:ov:ln:bo:ad	RAB1B	li:pe:ov:ad:lu	SLC1A1	pe:ov:li:ln:bo:lu	CMTM6	li:pe:ov:ad:lu
SNTA1	pe:li:ov:ln:bo:lu	A2M	pe:li:ov:bo:ad	ADH1A	pe:li:ln:ov:bo:ad	ADAMTS1	pe:li:ov:ln:ad
ZNF551	ov:li:pe:ln:ad:lu	SHBG	pe:ov:li:ad:lu:	CCL24	li:pe:ov:bo:ad:lu:	FNBP4	li:pe:ov:bo:ad:
GALNT10	pe:li:ov:ln:ad:lu	PLCL2	pe:li:ov:ad:lu	TGM1	li:pe:ov:ln:bo:lu	FOXA1	ov:li:pe:ad:lu
TAS2R4	pe:li:ov:bo:ad:lu	JPH2	li:pe:ov:bo:ad	CDCA3	li:ov:pe:bo:ad:lu	ZFYVE21	pe:ov:bo:ad:lu
SIRT4	pe:li:ov:ln:ad:lu	RBM7	pe:ov:ln:bo:ad	SPG21	li:pe:ov:ln:bo:ad	RBBP5	li:pe:ov:ln:lu
EIF2B5	pe:li:ov:ln:bo:ad	TAOK2	li:pe:bo:ad:lu	TSC22D4	pe:li:ov:bo:ad:lu	SERPINI2	ln:ov:bo:ad:lu
SON	li:pe:ov:ln:ad:lu	PPCDC	li:ov:pe:ad:lu	TRPC4AP	pe:ov:ln:bo:ad:lu	CD151	pe:li:ln:ov:ad
FLRT1	li:ov:pe:ln:bo:lu	TMOD2	pe:ov:bo:ad:lu	ZNF35	pe:li:ov:ln:bo:ad	ARFRP1	pe:li:ov:ad:lu
SP110	pe:li:ln:bo:ad:lu	XRCC1	pe:li:ov:ln:ad	CX3CL1	li:pe:ov:ln:bo:ad	AMELX	li:ln:ov:bo:lu
TREH	li:pe:ln:ov:bo:ad	CDS2	li:ov:pe:ln:bo	VGF	ov:pe:li:ln:ad:lu	HIST1H3E	li:pe:ov:bo:ad
SMO	pe:li:ov:bo:ad:lu	PRRX2	pe:li:ov:bo:ad	CDKL2	pe:ov:ln:bo:ad:lu	GIMAP6	pe:ov:ln:bo:ad
TIAM1	li:pe:ov:bo:ad:lu	TRIOBP	li:pe:ov:bo:lu	CYP46A1	li:pe:ov:ln:bo:ad	MTF2	pe:ov:li:bo:ad
ZKSCAN1	li:ov:ln:bo:ad:lu	DAK	li:pe:ov:bo:ad	CD160	li:ov:ln:pe:ad	XPO6	li:pe:ov:ln:bo
ARL4D	pe:li:ov:ln:bo:ad	PBXIP1	li:pe:ov:ad:lu	CHFR	pe:li:ov:bo:ad	CDK2	pe:li:ov:ad:lu
RIN1	pe:li:ov:ln:ad:lu	RNF10	li:pe:ov:bo:ad	TAF12	li:ov:bo:ad:lu	TACR3	pe:ov:bo:ad:lu
SALL1	pe:li:ov:ad:lu	RPL10A	li:pe:bo:ad:lu	ZNF667	li:pe:ov:ln:ad	ANKRD7	li:pe:ln:bo:ad
SLC9A3	li:pe:bo:ad:lu	PRDM14	pe:ov:li:ad:lu	SNCAIP	li:pe:ov:bo:lu	BPI	pe:ov:li:bo:ad
SOCS3	pe:ov:bo:ad:lu	RAP1A	li:pe:ov:bo:ad	MAS1	li:pe:ov:ln:bo	DOLK	pe:li:ov:ad:lu
G6PD	li:pe:bo:ad:lu	RHOF	pe:li:ov:ad:lu	RFK	li:pe:ov:ln:ad	ARL14	li:ln:ov:ad:lu
P2RX1	pe:li:ov:ad:lu	PLXNB1	pe:li:ov:ad:lu	GFOD1	li:pe:ov:bo:ad	NEIL3	li:pe:ln:ad:lu
CD84	pe:ln:bo:ad:lu	VEGFC	li:ov:pe:bo:ad	PRKD2	pe:ln:bo:ad:lu	TSGA10	li:pe:ov:bo:lu
SHANK1	pe:li:ov:ad:lu	TNS1	ov:pe:ln:ad:lu	ZFP36L1	li:ov:bo:ad:lu	PITPNA	li:ov:ln:bo:lu
TNKS	pe:li:ov:bo:ad	CUL1	li:pe:ov:ad:lu	AGL	pe:li:ov:ln:lu	PLS1	ov:pe:li:bo:ad
POLR3E	li:pe:ov:ad:lu	CFTR	pe:ov:li:ad:lu	ZFYVE16	li:pe:ln:ad:lu	CD9	li:pe:ln:ov:ad
PPP1R10	pe:ov:bo:ad:lu	ZFP64	pe:li:ov:ad:lu	TBC1D22A	ov:ln:bo:ad:lu	HIF1A	pe:li:ov:bo:ad
PIGA	li:pe:ov:bo:ad	SLC35A3	pe:ov:li:ln:bo	TMEM147	li:pe:ov:ln:lu	XBP1	pe:ov:ln:bo:ad
RREB1	li:pe:bo:ad:lu	DERL1	li:ov:ln:bo:ad	RENBP	li:ln:ov:bo:ad	ZDHHC14	pe:li:ov:bo:ad
TAF1	pe:ov:ln:bo:ad	PCSK1	li:pe:ov:bo:ad	FAM135A	li:pe:ov:ln:ad	GLRB	li:pe:ln:ad:lu
CIRBP	ov:ln:bo:ad:lu	SDAD1	li:pe:ov:ln:bo	SCD5	pe:li:bo:ad:lu	IER3IP1	pe:ln:bo:ad:lu
PRODH2	pe:ov:ln:bo:ad	RPL35	li:pe:ov:ad:lu	S100A1	ov:li:ln:bo:ad	TSHB	li:pe:ov:ad:lu
PFDN2	pe:ov:li:ln:ad	TEX2	pe:li:ov:bo:ad	LEMD3	pe:ov:li:bo:ad	GPR143	li:pe:ln:ov:bo
TECTA	li:ov:bo:ad:lu	STK3	pe:li:bo:ad:lu	CCRL2	pe:li:ov:bo:ad	SHC1	li:pe:ov:ln:bo
PNOC	li:pe:ov:bo:ad	RCN1	li:pe:ln:bo:ad	PDPN	pe:ov:bo:ad:lu	PAPSS2	li:ln:bo:ad:lu
USP4	pe:li:ln:bo:ad	PRDM1	pe:li:ln:bo:ad	GDPD3	li:pe:ov:ln:ad	SLC38A4	pe:ov:bo:ad:lu
CHN2	li:pe:ln:ad:lu	SFI1	li:pe:ov:ln:ad	SIL1	pe:ov:ln:bo:lu	ABCC2	li:pe:ov:ln:ad
SPAST	pe:li:bo:ad:lu	SNRK	ov:li:ln:ad:lu	IQSEC1	pe:ln:bo:ad:lu	MYOZ2	li:pe:ln:ov:bo
TULP2	pe:ov:li:ad:lu	OXCT2	li:pe:ov:bo:ad	TTYH1	li:ln:bo:ad:lu	BCL2A1	li:ov:pe:ln:ad
WNT1	li:ov:pe:ad:lu	P4HA1	pe:li:ln:ad:lu	SLC25A28	pe:ln:bo:ad:lu	ASL	li:pe:ov:ln:ad
MKNK2	li:pe:ov:bo:ad	DGKE	pe:li:ov:bo:ad	FZD6	ov:li:pe:ln:ad	GTF2A2	pe:ov:li:bo:ad
PLCD1	li:pe:ov:bo:ad	PIGC	li:pe:ov:ad:lu	IFT57	li:pe:ov:bo:lu	VPREB1	pe:li:ov:ad:lu
WNT11	pe:li:ov:bo:ad	SIVA1	li:pe:ov:bo:lu	ZNF638	li:ov:bo:ad:lu	CTSA	li:pe:ln:ad:lu
PGS1	pe:li:ov:bo:ad	SYNE2	pe:ov:bo:ad:lu	GRAP2	ov:li:ln:bo:ad	XPNPEP2	li:ln:ov:bo:lu
SPTLC1	pe:li:ov:bo:ad	SQRDL	li:pe:bo:ad:lu	MRPL52	ov:li:ln:bo:ad	SWAP70	li:pe:ov:bo:lu
PLA2G2A	li:pe:ov:bo:ad	GPX7	pe:li:ov:ad:lu	EXOSC2	pe:li:bo:ad:lu	TAF2	pe:ov:ln:ad:lu
SLC2A3	pe:ov:li:ln:ad	VILL	li:pe:ov:bo:ad	ZSCAN16	li:pe:ln:ad:lu	WT1	pe:ln:ov:bo:ad
SPEN	pe:li:ln:ad:lu	SNRPD1	li:pe:ov:ad:lu	LIF	li:ov:ln:bo:ad	ZNF281	pe:ov:ln:bo:ad
NOX4	li:ov:ln:ad:lu	TPM1	li:ov:bo:ad:lu	RRH	pe:li:ov:ln:bo	ZYX	pe:ov:bo:ad:lu
UBAP1	li:pe:ov:bo:ad	OBSCN	pe:ov:li:ad:lu	MFAP3L	pe:ov:bo:ad:lu	MPP1	li:pe:ov:bo:ad
ADA	li:pe:ov:bo:ad	SH3TC1	ov:pe:li:bo:ad	TTC22	li:pe:ln:ad:lu	MRPS22	li:pe:ov:bo:ad
TLE1	pe:li:ln:ad:lu	GZMH	pe:ov:li:bo:ad	ADAM18	li:ov:ln:bo:ad	LEFTY2	pe:ov:bo:ad:lu
RARRES2	li:pe:ov:bo:ad	EPN3	pe:ln:bo:ad:lu	CLIP4	li:ov:pe:ad:lu	CALML3	li:pe:ln:ad:lu
TBCA	li:pe:ov:bo:ad	ABAT	pe:li:ov:ln:bo	SPAG1	li:pe:ov:ln:bo	ZBED2	li:pe:ov:ln:bo
FADD	pe:ov:li:ln:ad	LTF	pe:li:ov:bo:ad	RPS27L	pe:ov:ln:bo:lu	SSX2IP	pe:ov:ln:bo:ad
CACNG3	pe:ov:ln:ad:lu	THUMPD2	li:ov:ln:ad:lu	NXT2	li:pe:ov:bo:lu		
ABCA4	pe:li:ln:ad:lu	FLNA	li:pe:ov:bo:ad	AUTS2	li:pe:ov:ln:ad		
YAP1	li:pe:ov:bo:ad	RPS6KA3	pe:li:ov:ad:lu	GK	pe:ov:bo:ad:lu		
		SLC2A11	li:ov:pe:ln:lu				

**(Left panel)** Genes significantly up-regulated (as described in Supplement 2) in the metastases over host tissue of at least 5 distinct target sites (of 7 sites total). **Right panel)** Genes significantly down-regulated in the metastases compared to host tissue of at least 5 distinct target sites (of 7). Liver = li, peritoneum = pe, ovary = ov, lymph node = ln, adrenal = ad, bone = bo, lung = lu.

**Figure 3:**
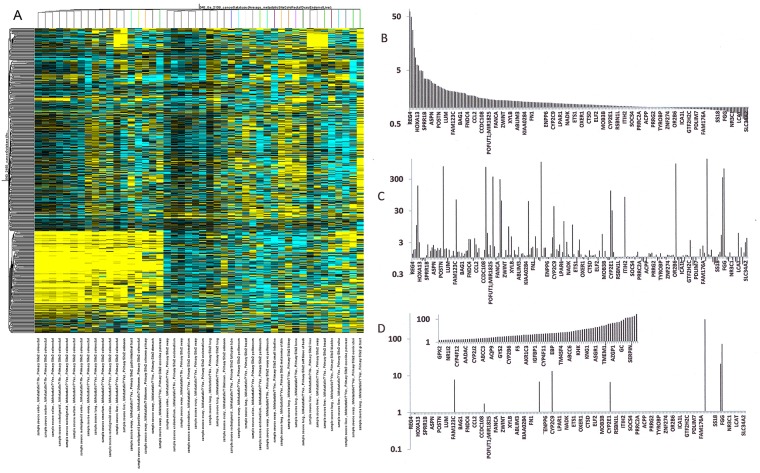
Comparison of the gene expression levels of primary colon cancers to colon cancer metastases in livers **(A)** Gene expression profiles among 47 diverse cancers. In the heat map of GSE2109, the colorectal cancers cluster, metastases by various primary tumors to the same organ also appear to share gene expression signatures. **(B, C)** The gene list is organized from high to low expression in the primary tumor according to GSE2109. The gene expression signatures of the metastases (C) are clearly distinct from the primary tumor (B). **(D)** Some up-regulated genes in the metastases (ASGR2, CRP, FGA, FGG, FGB, CYP2C9, ACSM2, ALB) are also high in healthy liver tissue. They may have been contributed by the host. However, the major expression profile in the liver (insert shows highly expressed genes in increasing order) is clearly distinct from the metastases, supporting the notion of a cancer-specific signature.

To extend the meta-analysis, we also investigated an individual GEO data set (GSE2109) that contains metastases from various primary cancer specimens. A comparison of 47 gene expression signatures reveals distinct patterns among primary and metastatic sites. Not only do the colorectal cancers cluster, metastases from various primary tumors to the same organ also appear to share gene expression signatures. Most liver metastases cluster together, so do most ovarian metastases, whereas the clustering of lung metastases is less tight (Figure 3A). Metastatic signatures were initially identified by comparing the gene expression levels in primary colon cancers (Figure 3B) to the gene expression levels of colon cancer metastases in the livers (Figure 3C). The gene regulation profiles of the metastases were clearly distinct from the primary tumor. It was possible that contamination from the host tissue (liver) accounted for the altered gene expression profiles. When comparing the metastases to intact liver, it was apparent that some up-regulated genes were likely contributed by the host tissue (ASGR2, CRP, FGA, FGG, FGB, CYP2C9, ACSM2, ALB) (Figure 3D). However, the predominant expression profile in the target organ is very different from the expression profile in the residing metastases. This supports the notion of a specific signature that characterizes metastases (Supplement 5).

### Metastases are distinct from their originating primary tumors through shared gene expression signatures

While cancers have lists for site-specific gene deregulation in their metastatic growths compared to their originating tumors (manuscript in preparation), the expression of some genes is altered in the metastases of all types of cancer, suggesting that these genes are generally essential for conveying invasive potential to a primary tumor (Table 2). Two genes (MAGEA12, TRPC7) are up-regulated and one gene (FCGRT) is down-regulated in the metastases from the 4 cancer types with significant gene lists. Multiple genes are deregulated in the metastases derived from 3 types of cancer. It may be important to note that the gene list in Table 2 may represent necessary contributors for metastasis initiation, while the gene list in Table 1 is likely reflective of metastasis outgrowth.

**Table 2:** Up- and down-regulated common genes in metastases compared to source tumor

UP				DOWN	
gene symbol	cancer source	gene symbol	cancer source	gene symbol	cancer source
MAGEA12	ov:br:pr:ki:	EIF3A	ov:br:ki:	FCGRT	ov:br:pr:ki:
TRPC7	ov:br:pr:ki:	TAX1BP3	ov:br:ki:	ERCC5	br:pr:ki:
BBOX1	br:pr:ki:	EYA3	br:pr:ki:	EPN2	br:pr:ki:
IPO9	br:pr:ki:	CTSE	br:pr:ki:	C6orf15	br:pr:ki:
TM6SF1	br:pr:ki:	MMP26	br:pr:ki:	NAALADL1	br:pr:ki:
HP1BP3	ov:br:ki:	LIG1	ov:br:ki:	SAMM50	br:pr:ki:
SUV420H1	br:st:ki:	TRIM28	br:pr:ki:	YIPF2	co:br:pr:
ZNF667	br:pr:ki:	CPS1	br:pr:ki:	CCDC85B	br:pr:ki:
CEP164	br:pr:ki:	ABCA2	ov:br:ki:	EGR4	br:pr:ki:
MAP2K4	br:pr:ki:	SEMA4A	br:pr:ki:	FBXO34	br:pr:ki:
POP4	br:pr:ki:	CNNM2	br:pr:ki:	CALCR	br:pr:ki:
SCGB1D2	br:pr:ki:	TLR2	ov:br:ki:	CAV2	br:pr:ki:
FARS2	ov:br:pr:	BRWD1	br:pr:ki:	KRT35	br:pr:ki:
DNAH7	ov:br:pr:	HPN	br:pr:ki:	S100A7	ov:pr:ki:
ZDHHC7	br:pr:ki:	RAC1	br:pr:ki:	TLE4	br:pr:ki:
TAP2	ov:br:pr:	DNAJB1	ov:br:pr:	LMO1	br:pr:ki:
GBE1	br:pr:ki:	EFHD1	br:pr:ki:	F9	br:pr:ki:
ZFP36L1	br:pr:ki:	DHX40	ov:pr:ki:	POGK	br:pr:ki:
FOXI1	br:pr:ki:	RAB26	br:pr:ki:	TRPV2	br:pr:ki:
ARMCX1	br:pr:ki:	ZNF639	br:pr:ki:	MLLT3	br:pr:ki:
C15orf39	br:pr:ki:	MYT1	br:pr:ki:	ZBTB6	br:pr:ki:
DDX17	br:pr:ki:	VAPA	ov:br:ki:	PTBP1	br:pr:ki:
DCHS1	br:pr:ki:	VPS39	ov:br:ki:	MCCC2	br:pr:ki:
POLD1	br:pr:ki:	SMPDL3A	br:pr:ki:	UBASH3A	br:pr:ki:
C1QA	br:pr:ki:	CRISP2	ov:br:pr:	CD163	br:pr:ki:
NARS2	ov:br:ki:	LRRC47	br:pr:ki:	GRIK1	br:pr:ki:
TNPO2	br:pr:ki:	NFIB	br:pr:ki:	SEC24C	br:pr:ki:
ATXN2L	br:pr:ki:	NCR1	br:pr:ki:	CCL8	br:pr:ki:
LYPLA1	ov:st:ki:	CPNE3	br:pr:ki:	ULBP2	br:pr:ki:
HRH4	br:pr:ki:	HECTD3	ov:br:ki:	RPL18	br:pr:ki:
NEIL3	br:pr:ki:	BRSK2	ov:br:pr:	COL17A1	br:pr:ki:
DHDDS	ov:br:ki:	SSTR1	br:pr:ki:	AOAH	br:pr:st:
GLP1R	ov:pr:ki:	COX11	br:pr:ki:	ATP1B3	br:pr:ki:
MVP	br:pr:ki:	CXCL6	br:pr:ki:	HAPLN1	br:pr:ki:
SCLY	br:pr:ki:	LTA	br:pr:ki:	MLC1	br:pr:ki:
MAPK6	br:pr:ki:	CASK	br:pr:ki:	ELOVL5	br:pr:ki:
C1QB	br:pr:ki:	HIPK1	ov:br:pr:	PARP16	br:pr:ki:
ZNF34	br:pr:ki:			FIGF	br:pr:ki:
SLC39A2	br:pr:ki:			B3GALT2	br:pr:ki:
ZFAND3	br:pr:ki:			PDCD1	br:pr:ki:
SNX24	ov:pr:ki:			RGS17	br:pr:ki:
TDO2	br:pr:ki:			TCN2	br:pr:ki:
ADCY7	ov:br:ki:			RBM28	br:pr:ki:
LPO	br:pr:ki:			ARHGAP11A	br:pr:st:
CCDC64	br:pr:ki:			TGM2	br:pr:ki:
KRT1	br:pr:ki:			SEMA3E	br:pr:ki:
SDAD1	br:pr:ki:			TNFSF10	br:pr:ki:
MSMB	br:pr:ki:			CASD1	br:pr:ki:
GDAP1L1	br:pr:ki:			SF3A2	br:pr:ki:
SULT1C2	br:pr:ki:			TPM1	br:pr:ki:
KCND3	br:pr:ki:			SLC18A2	br:pr:st:
ENO2	br:pr:ki:			CACNG3	br:pr:ki:
BMP2	ov:br:ki:			MICB	br:pr:ki:
ZBTB24	ov:br:pr:			DNAJB9	br:pr:ki:
FHOD3	br:pr:ki:			WDHD1	br:pr:ki:
DMXL2	ov:br:ki:			PSMB1	br:pr:ki:
EID1	br:pr:ki:			CAPN7	br:pr:ki:

**(Left panel)** Genes significantly upregulated (as described in Supplement 3) in the metastases over primary tumors of at least 3 distinct types of cancer. **Right panel)** Genes significantly downregulated in the metastases over primary tumors of at least 3 distinct types of cancer. Ovary = ov, breast = br, prostate = pr, kidney = ki, stomach = st, colon = co.

### A murine metastasis model corroborates the meta-analysis results

**Figure 4:**
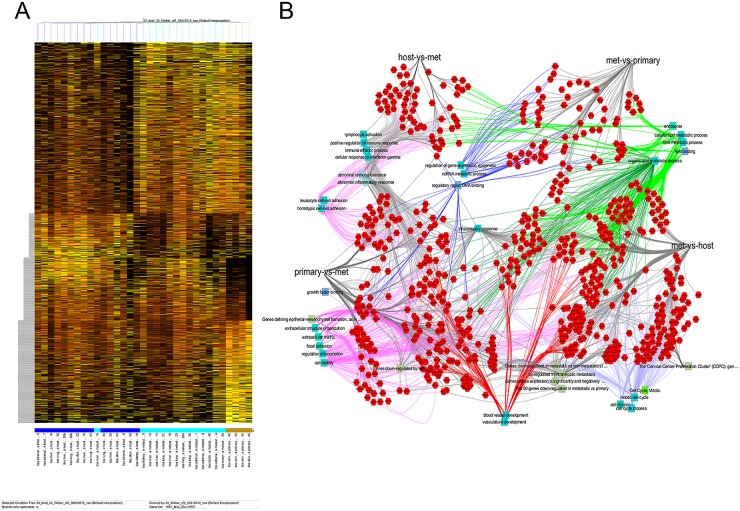
Murine metastases B16-F10 cells were injected intravenously and disseminated colonies were retrieved after two weeks for cell sorting and ensuing RNA extraction. Site- and tissue-specific gene expression was assessed by RNAseq. **(A)** Heat map of gene expression comparing host tissue to metastases to primary (undisseminated) tumor. **(B)** Network map (generated with Cytoscape 3.5.1) indicating the relationships among differentially expressed genes in metastases, cognate host tissue and cutaneous melanoma. The corners contain the comparisons of host versus metastases (top left), primary tumor versus metastases (bottom left), metastases versus host (lower right), and metastases versus primary tumor (top right). Connectivity related to metabolism (top right) is shown with dark or light green edges, vascularization (bottom) has red edges, and cell motility/cell adhesion (lower left) are connected via lilac edges.

We tested the meta-analysis results experimentally, using the cell line B16-F10. After tail vein injection, it is metastatic to multiple sites, and the subcutaneous injection approximates a primary tumor. The comparison of B16-F10 metastases to host tissue and primary tumor reveals groups of up- or down-regulated genes in all pairwise comparisons (Figure 4A). A network map shows the importance of metabolism, vascularization and extracellular matrix interactions (Figure 4B). As had been the case in the meta-analysis of human data, the GO categories for biological function by the murine metastases displayed the frequent representation of skewed metabolism, matrix interaction/migration, vascularization, and alteration in ionic homeostasis – for the comparison of metastases to their cognate host site as well as metastases to the primary tumor (Supplement 6). When comparing to the core metastasis signature identified in the meta-analysis (genes up- or down-regulated in most or all metastases in relation to either their primary tumor or the target host tissue) a large proportion of the identified human metastasis genes also display similar expression changes in the murine metastases.

## DISCUSSION

The genetic core program of metastasis encodes four functional entities, which are activated over the primary tumor and are maintained in the target site. Firstly, the gene expression analysis corroborates recent findings that metastasis genes alter the metabolism of cancer cells to increase energy production [35]. This adjustment conveys anchorage-independent survival [36, 37], a feature that may still be required in the foreign micro-environment of a metastatic target organ [38]. Secondly, angiogenesis and tissue remodeling is also reflected in the gene expression profiles. Tissue remodeling is consistent with our recognition that metastasis genes are stress response genes [7, 8]. A third component is involved in reducing extracellular matrix interactions and enhancing cell motility. This reflects the feature of cancer cell homing. As the number of genes down-regulated in metastases compared to primary tumors is substantial, the inactivation of metastasis suppressor genes may be as important for cancer dissemination as the up-regulation of metastasis genes [34, 39, 40]. Down-regulation was previously observed for bone marrow micro-metastases of breast cancers [41]. Fourthly, we found in a prior study that anchorage-independence is associated with genetic reprogramming for the homeostasis of inorganic ions [42]. This phenomenon seems to play a continued and substantial role in the destination sites of dissemination. Inorganic molecules, including copper and potassium, have been associated with tumor progression and angiogenesis [43–45]. The molecular connections, patho-physiologic effects and therapeutic targeting potential of the metastasis-associated modifications in ionic balance remain to be fully elucidated.

In various target organs, the four core modules are activated through overlapping, but topologically distinct genetically encoded programs. Beside the four required functional entities, adjustments to the particular host tissue micro-environment, which has been colonized, are also important for the outgrowth of clinically relevant cancer metastases. These adaptations reflect the site-specific component of the genetic metastasis signature. The gene expression profile of metastasis is characterized by a core program component that increases motility/metabolism/vascularization/ionic balance plus a specific program component that adjusts to the target site.

Cancer dissemination is associated with alterations in gene expression within the cancer cells. The metastasis gene deregulation is not specific for any primary tumor. This has been known for individual metastasis genes, such as osteopontin, which is associated with the progression of about 30 different cancers [46, 47]. We now expand this paradigm to entire gene expression profiles, as we have found common signatures for metastases that are not active in the original type of cancer (see Table 2) and signatures that are shared in metastases to the same organ (see Table 1).

Gene expression analysis cannot discern whether the RNA profile of a metastasizing cancer cell is altered at the onset of dissemination, and thus is the determinant of organ preference, or is shaped in the metastatic micro-environment, and thus is a consequence of having arrived at a specific target organ (the question is under active discussion [48–50]). Frequently, studies into cancer aggressiveness have focused on biomarkers in the primary tumors. The over-expression of specific genes has been associated with risk for progression in numerous types of cancer. Remarkably, our analysis finds several genes with such attributions, including metalloproteinases (specifically MMP1), chemokines, and POSTN to be suppressed in disseminated growths. This suggests that some of the gene products for tumor progression are only required for the early stages of cancer spread, not for the outgrowth of distant metastases after the transformed cells have reached their target organs. The disappointing clinical trial results with MMP inhibitors may well indicate that the drugs were given too late in disease progression [51].

The meta-analysis approach to the evaluation of actual patient data escapes the compromises associated with small local cohorts in original studies or with *in vivo* experimental models for cancer, but it is limited by the correlative nature of the results obtained. The robustness of the findings is also dependent on quality and quantity of the input data sets, which vary with source and target sites. Derived from increasingly available computing power and cross-platform normalization algorithms, the ability to assemble multi-gene lists for distinguishing metastases from their primary growths and mapping them to their target organs has identified gene regulation pathways, some of which have thus far escaped laboratory research. Such pathways may be detectable only in their actual molecular pathophysiology context, but not in experimental research models that selectively target individual genes. While requiring confirmation through additional research approaches, the investigation of 8723 genes in 653 gene expression profiles (close to 5.7 million data points) has generated novel insights. Confirmation has been obtained in a murine model of metastasis.

The treatment of metastatic cancer has historically been guided by the originating primary tumor, such that liver metastases from colorectal cancer have received chemotherapy regimens deemed appropriate for primary colorectal cancer whereas liver metastases from pancreatic cancers have been treated with the drug combinations found suitable for cancers originating in the pancreas. Because the gene expression signatures of cancer metastases change substantially from their primary tumors and because metastases from various anatomical sites of origin to the same target organ converge in their gene expression patterns, a more promising strategy could be to focus the choice of combination chemotherapy on the target organ for metastases. This would imply that it may be more efficacious to treat all liver metastases with similar drug regimens that target the genetic core program of metastasis or the site-specific genetic program of liver metastasis, regardless of their organ of origin.

## MATERIALS AND METHODS

### Meta-analysis

### Source data

We investigated the gene expression arrays from a search for solid tumor metastases, comprising a total of 653 metastases and their controls. All array data and their annotations were downloaded from NCBI's GEO database [52]. Of these 653 mined experiments, 449 had been performed using the Affymetrix platform, and the remaining 204 had utilized the Agilent platform. These samples belong to 8 “same target site” groups and 6 “same source site” groups, where the former refer to specimens derived from different primary tumor sites but metastasized to the same target locations, while the latter refer to specimens with the same primary tumor locations and metastases into various target organs. The “same target site” group includes 294 samples. The “same source site” group includes 551 samples. 192 samples are shared between the “same target site” and the “same source site” groups (Supplement 1). The target site “chestwall” was not sufficiently defined for matching host tissue to be identified, so it was included only in the “same source site” analysis. The meta-analysis was conducted by AccuraScience.

### Informatics reconciliation among array platforms and versions

The entire set of array data was derived from 5 different versions of Affymetrix arrays and 1 version of Agilent arrays. Because this study focused on coding RNA and aimed at covering all GEO sets identified in the search, all platforms were included. The source heterogeneity posed two primary challenges for an integrated analysis of array data across different platforms and their multiple versions. The first challenge was an informatics task involving the integration and reconciliation of annotation data from the diverse array platforms and versions. This was handled with a Perl program (Supplement 1). The second challenge was to identify a proper method for removing the batch effect, and to perform proper cross-platform normalization of these array data from various origins. YuGene [53] uses a cumulative proportion transform. Let *P_i_* denote the expression of a gene, and *P_(i)_* denote the expression of the same gene, but in descending order. The YuGene transformed value for the gene is

$$\begin{array}{lll} Y_{\left(i\right)} & = & 1-\frac{\sum_{j=1}^{i}P_{\left(j\right)}}{\sum_{j=1}^{p}P_{\left(j\right)}}=\frac{\sum_{j=1}^{p}P_{\left(j\right)}-\sum_{j=1}^{i}P_{\left(j\right)}}{S_{p}}\\ & = & \frac{{\sum_{j=i+1}^{p}P}_{\left(j\right)}}{S_{p}}for\;i=1,\ldots ,p-1\\ Y_{\left(P\right)} & = & 0. \end{array}$$

For most data sets, this process achieved excellent normalization (see Supplement 1).

### Principal component analysis

Principal component analysis (PCA) allows all samples to be examined on a low-dimensional space, representing the first few principal components. It also offers an effective way to evaluate and compare normalization methods. PCA was carried out using the function *pca()* in the R (version 3.2.1) package *FactoMineR* (version 1.31.4) [54], using all genes shared among all array platforms/versions. Configuration: scale.unit = TRUE, ncp = 5, ind.sup = NULL, quanti.sup = NULL, quali.sup = NULL, row.w = NULL, col.w = NULL, graph = TRUE, axes = c(1,2). PCA was done separately for the gene expression profiles in the “same target site” groups and the “same source site” groups, following YuGene-based normalization. The results allowed for a few clearly distinguishable clusters to be identified.

### Differential expression analysis

Differential expression analysis of the array data was accomplished using the *limma* R package [55]. For each of the “same source site” groups, comparison was made between the primary tumor growth (representing the source site), and all metastases (regardless of their target sites) lumped together. For each of the “same target site” samples, comparison was made between the healthy host organ (the target site) and tissue-specific metastases to that site from all source tumors lumped together. Multiple stringency cut-offs (FDR < 0.05, FDR < 0.2 and P value < 0.05) were used to select significantly up- and down-regulated genes.

A second approach conducted pair-wise differential expression analysis between each of the metastatic sites and the primary source tumor, or between the healthy target site and each of the metastases from various primary tumors to that site. Following a non-stringent cut-off, the obtained gene lists were included in the pathway enrichment analyses. This procedure of pairwise evaluation resulted in identical gene lists and GO category lists to the lumped analysis, differing only in stringency (the number of members on the lists). Whether the source sites were lumped or compared pairwise to the metastatic target sites, the smaller of the resulting gene lists/GO categories was always contained in the larger list (with 0-7% of the entries in the shorter list diverging from the longer list, not shown). Hence, we performed the study with lumping together metastases in “same target site” or primary tumors in “same source site” investigations.

### Pathway enrichment analysis

Differentially expressed genes were used as input for the pathway enrichment analysis, which identifies biological pathways (denoted as Gene Ontology terms or GO categories) that are associated with the up-regulated or the down-regulated genes. The function *enrichDAVID()* in the R (version 3.2.1) package *clusterProfiler* (version 3.0.1) [56] was utilized. Because the maximal number of genes that *clusterProfiler* can accept as input is 2000, when the number of up- and down-regulated genes was greater only 2000 genes were randomly selected. An FDR cut-off of 0.05 was used in selecting significant pathways. Enriched categories (GO terms) were evaluated with the understanding that these results are highly susceptible to many factors. The P-value of a GO term was produced using Fisher's exact test (a hypergeometric distribution-based test), which is very sensitive to the numbers of genes in the up- or down-regulated gene list.

### Individual data sets

To identify metastatic signatures, we compared the gene expression levels in primary colon cancers with the gene expression levels in colon cancer metastases to the liver. The source data comprise a human data set from GEO (GSE2109). An assessment of 47 signatures, derived from various specimens, reflected on gene expression patterns among primary and metastatic sites. It also allowed the comparison between metastases and healthy target tissues. To identify the gene expression signature of metastases to the same organ from various primary tumors, we analyzed the expression profiles of liver metastases (from cancers of the breast, colorectum, exocrine pancreas, ovary, vulva, and unknown site; each corrected for contamination by the host tissue) for genes with the least variation in expression. This approach was based on the rationale that the highly consistently expressed genes are most likely to be functionally important in organ-specific metastasis, whereas large variations in expression levels may reflect noise generated by the various organs of origin of the primary tumors or by inter-individual differences among patients. Approaches that consider signal-to-noise metrics have been used successfully to analyze metastases of diverse origins [33].

We analyzed a human breast cancer Agilent data set in GEO (GSE26338-GPL1390) [57]. From 22576 genes, we identified 687 with location-specific expression patterns by using ANOVA (with equal or non-equal variance assumptions) with Benjamini-Hochberg FDR < 0.05, then combined the total of 1146 genes to seek expression differences above 25%. The results were clustered and divided into 11 k-means groups for the identification of differentially expressed genes. The data were treated with rows (genes, those with few probes averaged to one value) and columns aligned. This first run had grouped the columns according to breast tumor and then the different sites of metastasis.

### Multi-pathway analysis

We have brought into production a new web server, http://metastatic.cchmc.org, in which we have gathered and combined a great deal of current reference knowledge and data derived from the literature and from published genomic data sets. The system includes the ability to search for metastatic cancer-related information derived from disease descriptions in OMIM, UMLSKS, mouse knockout and transgenic allele phenotypes, gene ontology and pathway data sources, and gene expression, epigenetic, and chemical biology-based genomic data sets that relate to metastatic experimental models, phenotypes, or observational profiling studies. The tool allows for subsequent integration of this information and the construction of biological networks [58]. Using a database constructed from gene expression microarray data of cancer samples (http://gataca.cchmc.org/gataca/metastatic), we now analyze differential gene expression in distinct metastatic tumors and metastatic sites.

### Murine metastasis model

The *in vivo* experiments were conducted under an IACUC-approved protocol, which followed the standards of the ARRIVE guidelines [59]. The B16-F10 cells were obtained from ATCC. Cells were kept in culture for no more than 4-6 weeks before replacing with a fresh batch. Mycoplasma tests were conducted on a regular basis (every 4-8 weeks). We injected C57Bl/6 TG [UBC-GFP] mice with 0.2 × 10^6^ B16-F10 dsRed melanoma cells via the tail vein. This allowed the formation of disseminated foci without the presence of large primary tumors requiring early termination of the experiment. While pulmonary dissemination was predominant, metastases were found in multiple organ sites. Subcutaneously injected cells (rear flank) served as model for the primary tumor. After 14 days, total necropsy identified metastases as black spots on the surface of target organs (lungs, liver, adrenals, kidney, ovaries, testes). The spots were excised with a narrow margin, single cell suspensions were generated by grinding between frosted glass slides, followed by passing through a strainer. The cells were sorted according to forward scatter, side scatter, red and green fluorescence in order to separate metastatic cells from host cells.

RNA was extracted using the Qiagen RNAeasy kit. An initial amplification step comprised the use of the Ovation RNA-Seq System v2 (NuGEN) to create double-stranded cDNA from 0.5 ng total RNA. The concentrations were calculated using the Qubit dsDNA BR assay. An Agilent RNA Nano 6000 LabChip analyzed the size distribution, and the samples confirmed the expected size distribution traces. Nextgen sequencing was performed on SE100 at 20 million reads per sample, covering 36,000 genes.

## SUPPLEMENTARY MATERIALS FIGURES AND TABLES














