# Supplementary material for: A core program of gene expression characterizes cancer metastases
Source: Oncotarget. 2017 Nov 2;8(60):102161–75. doi: 10.18632/oncotarget.22240 (PMC5731943; doi:10.18632/oncotarget.22240)
Supplement: Supplementary file 1 [file oncotarget-08-102161-s001.pdf]

A core program of gene expression characterizes cancer metastases

SUPPLEMENTARY MATERIALS

Supplement 1: GEO data sets and normalization

We searched the GEO database for solid tumor metastases and downloaded all hits (Supplementary Tables 1.1.A and B). We conducted an updated literature search and determined that YuGene, ComBat and SCAN were among the current methods most promising for batch effect removal from profiles that comprise both Affymetrix and Agilent data. SCAN (Piccolo SR, Withers MR, Francis OE, Bild AH, Johnson WE. Multiplatform single-sample estimates of transcriptional activation. Proc Natl Acad Sci 2013;110:17778-17783.), though it ran flawlessly with all Affymetrix data and tested well with the test Agilent data set that is provided with the program, failed to open the Agilent data of this project (likely due to a change of Agilent's data format that took place after the latest version of SCAN was developed). We were unable to overcome this problem, and had to abandon SCAN from further consideration. The principal component analysis (PCA) results following ComBat-based normalization (Chen C, Grennan K, Badner J, Zhang D, Gershon E, Jin L, et al. Removing batch effects in analysis of expression microarray data: an evaluation of six batch adjustment methods. PloS One 2011;6:e17238.) reflected an artifact. Many samples were closely aligned along the vertical axis, suggesting that PC1 failed to differentiate (the samples with breast as source site spread out and spanned a large range; all samples with other source site groups were clustered in a very small range). Close examination indicated that those samples with breast as the source site were all Agilent-based, in contrast the other samples were all Affymetrix-based. These observations are consistent with reports from the literature that, although ComBat is able to handle both Affymetrix and Agilent data and it can remove batch effects reasonably well for either platform, this method does not perform well for cross-platform normalization. We decided not to consider ComBat-based normalization in this project. YuGene uses a cumulative proportion transform and was applied to the task. For most data sets, this process achieved excellent normalization. Only a small number of GEO gene expression profiles clustered away from the signatures of other profiles in the same group. They turned out to reflect batch effects from select sample deposits. The affected GSM numbers were judged as not having been normalized properly with YuGene and were excluded from analysis (Supplementary Tables 1.1.C and 1.1.D).

A Perl program (AccuraScience) developed for this project to process and reconcile the probe annotation files for each of these array versions, using which, we were able to extract 8,723 genes that are shared among the relevant array types (Supplementary Table 1.2.). All other genes were eliminated from the downstream analysis. The array data were then reprocessed, whereby the expression levels of multiple probes corresponding to the same gene were averaged to represent the expression level of the gene.

Supplementary Table 1.1: GEO data sets

A Summary of sample numbers in the "same source site" groups. **B** Summary of sample numbers in the "same target site" groups. **C** Detailed listing of sample information for "same source site" group. **D** Detailed listing of sample information for "same target site" group. The strikethroughs in sections **C** and **D** indicate data sets that could not be normalized with YuGene and were eliminated from further consideration as confirmed outliers.

Supplementary Table 1.2: Summary of probe and gene information

Several array platforms and versions are associated with the GEO data sets involved in this study.

See Supplementary File 1

Supplement 2: Metastases by target site

Up-regulated genes identify those with higher expression levels in the metastasis samples as compared to the healthy host tissues, down-regulated genes are lower in the metastases than in the host tissue. Select genes are up- or down- regulated in all or most of the target sites analyzed (Supplementary Table 2.1). In addition, metastases to specific target sites have unique gene expression signatures (Supplementary Table 2.2). To rationalize the correlative meta-analysis results, we followed up with an in silico analysis of likely interactions between the gene products induced in lymph node metastases and those highly expressed in host lymph nodes. Multiple potential interactions were identified (Supplementary Figure 2.1).

Supplementary Table 2.1: Significantly up- and down-regulated genes by target site

Summary of differentially expressed genes in metastases over their individual target sites and significantly enriched pathways. Cut-offs of FDR<0.05 or P<0.05 were used in selecting significant pathways.

Supplementary Table 2.2: Gene ontology pathways altered in various metastatic sites

Top 50 changes in gene expression pathways within the groups of metastases compared to the host organ. For lists that have more than 50 significant results, only the starkest changes, according to smallest false discovery rate (FDR), are presented. Gene ontology categories in bold/underline reflect components of the intermediary metabolism, bold indicates vascular changes, underline is associated with cell movement or adhesion, italics stand for inorganic ion transport and homeostasis. These four functional groups comprise about one fourth (25.54%) of the top gene ontology category changes compared to the host organ (liver 19 up/29 down, lung 25/35, bone 37/21, ovary 19/23, peritoneum 24/38, adrenal 17/21, lymph node 24 down of the 100 most affected GO categories). **A)** Enriched pathways among up-regulated genes for the same target group. **B)** Enriched pathways among down-regulated genes for the same target group.

See Supplementary File 2

Supplement 3: Metastases by origin

Supplementary Table 3.1: same source summary

Summary of significantly up-regulated and down-regulated genes in metastases (to various sites) derived from the same tumor, and resulting significantly enriched pathways at FDR<0.05 or P<0.05. Up-regulated genes refer to genes with higher expression levels in the metastasis samples as compared to the primary tumor tissues. The number of input data sets varied substantially among cancers (compare Table 1.1B).

Supplementary Table 3.2: Significantly up- or down-regulated pathways in metastases compared to source tumor

Enriched pathways among up-regulated or down-regulated genes in the same source group. Gene ontology categories in bold/underline reflect components of the intermediary metabolism, bold indicates vascular changes, underline is associated with cell movement or adhesion, italics stand for inorganic ion transport and homeostasis. These four categories comprise about one fourth (25.67%) of the top changes in metastases compared to their primary tumors (breast 27 up/32 down, kidney 37/19, prostate 13/26). Upregulated in all 4 groups is GO:0032268 (regulation of cellular protein metabolic process). Upregulated in 3 groups are GO:0008283 (cell proliferation) and GO:0006928 (cell motion). Downregulated in 3 groups are GO:0051094 (positive regulation of developmental process), GO:0042803 (protein homodimerization activity). **A)** Upregulated GO categories. **B)** Downregulated GO categories.

See Supplementary File 3

Supplement 4: Breast cancer metastases

We compared profiles from primary breast cancers with profiles from either healthy breasts or metastases (GSE26338) (41). Expectedly, the breast cancers have a pattern of significantly expressed genes that is distinct from healthy breasts (Supplementary Figure 4.1A-C). We also identified the metastatic breast cancer gene expression signature by comparing the breast cancers to their matched metastases. Metastases (to all sites combined) display distinct gene expression profiles compared to the primary tumors. Gene ontology analysis identified functional categories associated with oxidative metabolism and vasculature to be activated and genetic programs for extracellular matrix interactions to be down-regulated in metastases (Supplementary Table 4.1A). In breast cancer metastases, there are more genes downregulated (including POSTN, LUM, ASPN, COL12A1, MXRA5, FBN1) than are up-regulated (including PCSK4, DLK1, SAPS1, ANGPTL4) compared to the primary tumor (Supplementary Table 4.1B). We then addressed differences among distant breast cancer growths in various organs. The metastases to lymph nodes, liver, lungs, skin, and brain displayed gene expression signatures that were mutually distinct and were different from the primary tumor, implying that the pattern of gene expression may identify a metastatic site (Supplementary Figure 4.2). While some genes are significantly (P<0.01) up- or down-regulated in multiple sites (Supplementary Table 4.2A) others contribute to the organ-specific signatures (Supplementary Table 4.2B). The downregulation of MMPs in various metastatic sites is striking. This may reflect a role in shedding from the primary tumor, but not in implantation at the target site. Of note, MMP1 and MMP2 have been previously described to be under-expressed in liver metastasis compared with primary colorectal cancers (16).

Supplementary Table 4.1: Gene expression changes in breast cancer metastases

Metastases in GSE26338 to all sites were grouped and compared to the primary breast cancers. **A)** Gene ontology and other categories associated with metastasis. **B)** Genes up-regulated or down-regulated in metastases compared to the primary tumors (two distinct analysis algorithms arriving at similar gene lists – only one result is shown).

Supplementary Table 4.2: Gene expression changes in breast cancer metastases to specific sites

The normalized expression levels of the distinct metastatic sites in GSE26338 were compared to the normalized expression levels of the primary breast cancers that had matched metastases. **A)** Genes that are significantly ($p < 0.01$) up- or down-regulated in multiple sites. **B)** Shown are the 20 most up-regulated genes and the 20 most down-regulated genes for each site.

See Supplementary File 4

Supplement 5: Liver metastases

We hypothesized that metastases to the same organ from various primary tumors would be characterized by shared gene expression signatures. To identify those, we analyzed the expression profiles of liver metastases (from cancers of the breast, colorectum, exocrine pancreas, ovary, vulva, and unknown site; each corrected for contamination by the host tissue) for genes with the least variation in expression. This approach was based on the rationale that the highly consistently expressed genes are most likely to be functionally important in organ-specific metastasis, whereas large variations in expression levels may reflect noise generated by the various organs of origin of the primary tumors or by inter-individual differences among patients. We identified a set of 53 genes, the expression of which is shared among liver metastases derived from various origins (Supplementary Table 5A). Some of these liver metastasis genes are dramatically over-expressed (up to 40-fold). 35 of them are also present in the list of significantly deregulated genes in the meta-analysis of liver metastases (see Supplementary Table 2.2A, B). We followed up with gene ontology analysis of these most highly affected genes in liver metastases from diverse cancers. The top 30 GO categories, according to the lowest p-values, comprise genes of extracellular regulation/cell motility, tissue remodeling/angiogenesis, and intermediary metabolism. Categories of transmembrane ion transport are represented as well (Supplementary Table 5B). 15 of these GO categories are also present in the meta-analysis list of GO categories for liver metastases. The results corroborate that the pattern of metastasis gene expression is not shaped by the site of origin of any primary tumor, but reflects core program of metastasis plus unique characteristics of the particular target organ.

The gataca software allows an analysis of pathways, co-expression, and transcriptional co-regulation in biological network plots. We identified principal pathways and functions that are specific or shared between the two different signature gene lists of GSE2109 colorectal cancer metastases to the liver or lungs respectively (Supplementary Figure 5).

Supplementary Table 5: Gene expression signatures of cancer metastases to the liver

The data set GSE2109 provides information on metastases from various sites of origin. **A)** The gene expression profiles of liver metastases from various cancers (breast, colorectal, exocrine pancreas, ovary, vulva, unknown site; corrected for contamination by the host tissue) were analyzed for genes with the least variation in expression (lowest standard deviation as percent of the mean among the samples and ratios among all the specimens closest to 1, both measures gave identical results). To focus on genes that are likely to substantially impact biological function, we limited the search to normalized expression levels above 2. This identified a set of 53 genes, the expression of which is shared among metastases of various origins. B) Gene categories expressed in liver metastases. Gene ontology analysis of the most highly expressed genes in liver metastases from various cancers, after correction for contaminating host tissue. In liver metastases, the top 30 GO categories, according to the lowest p-values, comprise genes of extracellular regulation/cell motility, tissue remodeling/angiogenesis, genes that regulate the intermediate metabolism and anion transport. Also present in the meta-analysis list of GO categories for liver metastases are GO:0001568 (blood vessel development), GO:0001944 (vasculature development), GO:0048514 (blood vessel morphogenesis), GO:0001525 (angiogenesis), GO:0016323 (basolateral plasma membrane), GO:0016477 (cell migration), GO:0051674 (localization of cell), GO:0048870 (cell motility), GO:0016820 (hydrolase activity, acting on acid anhydrides, catalyzing transmembrane movement of substances), GO:0008509 (anion transmembrane transporter activity), GO:0006820 (anion transport), GO:0004252 (serine-type endopeptidase activity), GO:0008236 (serine-type peptidase activity), GO:0017171 (serine hydrolase activity), and GO:0007586 (digestion).

See Supplementary File 5

Supplement 6: Murine metastases

Supplementary Table 6.1: Gene expression signatures in murine metastases

The most significantly deregulated GO categories for biological function in B16-F10 metastases (shown are the top 50 according to lowest p-values and FDR). The two left panels compare metastases to the primary tumor, while the two right panels depict the comparison of metastases to their cognate host sites. Gene ontology categories in bold/underline reflect components of the intermediary metabolism, bold indicates vascular changes, underline is associated with cell movement or adhesion, italics stand for inorganic ion transport and homeostasis.

Supplementary Table 6.2: Gene expression signatures in murine metastases

A) Genes of the human core signature for metastasis (compared to the host tissues – see Table 1), which are also altered (up at least 2-fold, down at least 0.5-fold) in the murine model. **B)** Genes of the human core signature for metastasis (compared to the primary tumor – see Table 2), which are also altered (up at least 2-fold, down at least 0.5-fold) in the murine model.

See Supplementary File 6.

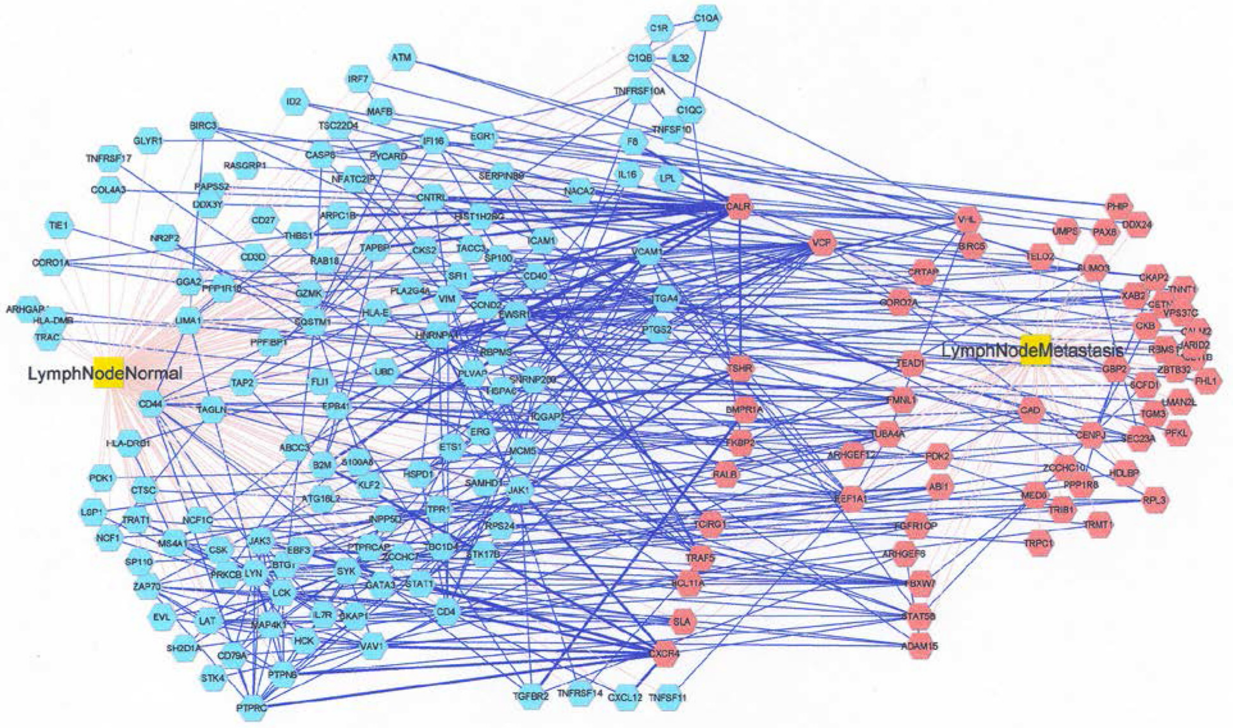

Supplementary Figure 2.1: Tumor Host Interactome Analysis to prioritize candidate interactions between genes strongly expressed in lymph node metastases (nodes in red) and host lymph nodes (nodes in turquoise). The gene expression signature for healthy lymph node was obtained from Toppgene gene expression atlas module data derived from the Immunogenome Consortium. Protein-protein interaction analysis was performed using ToppCluster.cchmc.org of strongest-expressed genes associated with functional categories of secreted, cell surface, and extracellular matrix genes that belong to categories including growth factors, receptors, and others, which have the potential to reflect regulatory matrix and receptor-ligand interactions between metastasis and host.

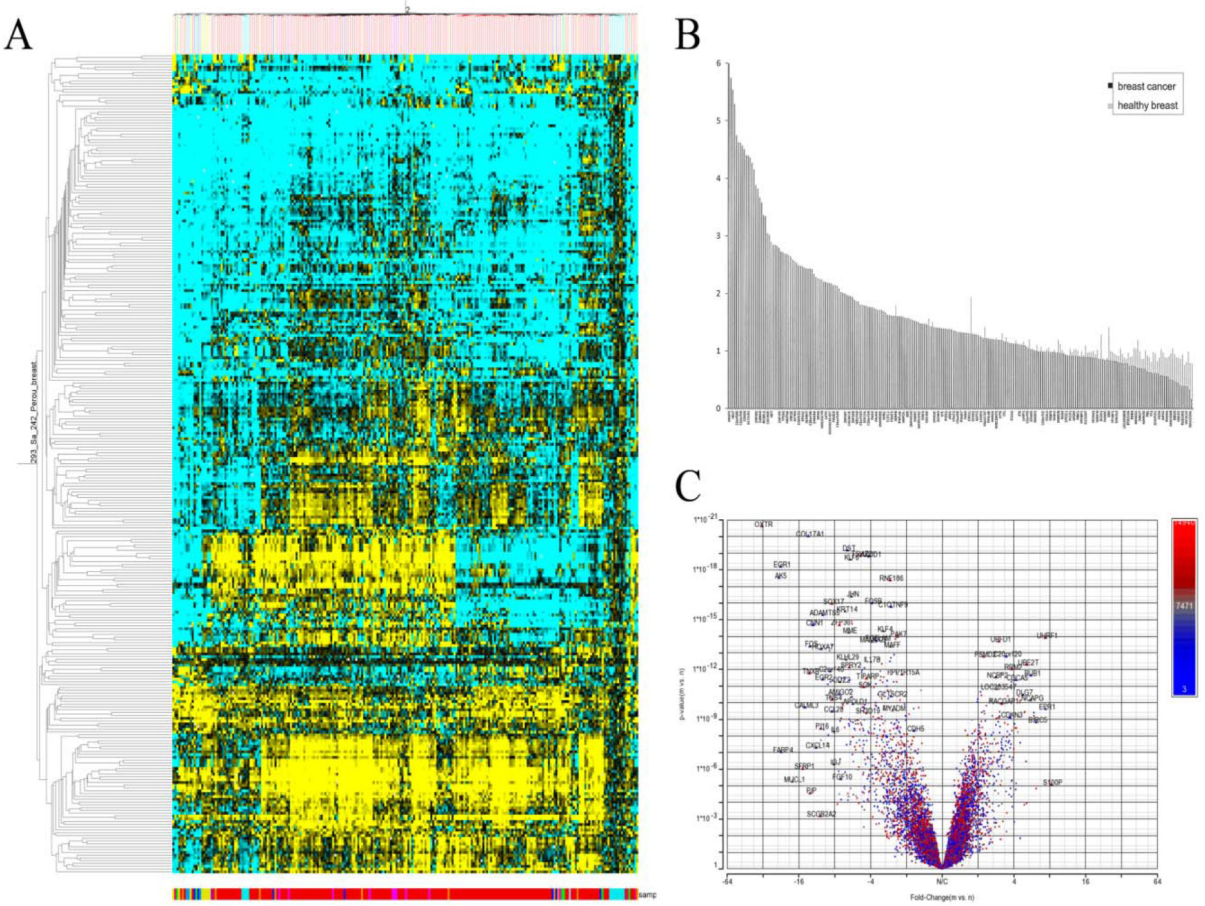

Supplementary Figure 4.1: Gene expression in breast cancer versus healthy breasts. The data set GSE26338 provides information on breast cancer. **(A)** Heat map of all breast cancers (n = 181) versus healthy breasts (N = 12). **(B)** Gene expression differences between metastatic breast cancers (n = 7, cancers with matched metastases in the same data set) and healthy breasts (n = 12). The y-axis shows relative expression levels for differentially expressed genes with p-values below 0.05. **(C)** Volcano graphs convey graphically the most significant genes in a comparison between the 2 groups. The x-axis represents the fold change in gene expression levels. The center is 0 (no change), right is the direction for up-regulation and left for down-regulation in breast cancer compared to healthy breasts. The y-axis indicates the p-value. Some of the genes with the greatest differences are labeled in this graph.

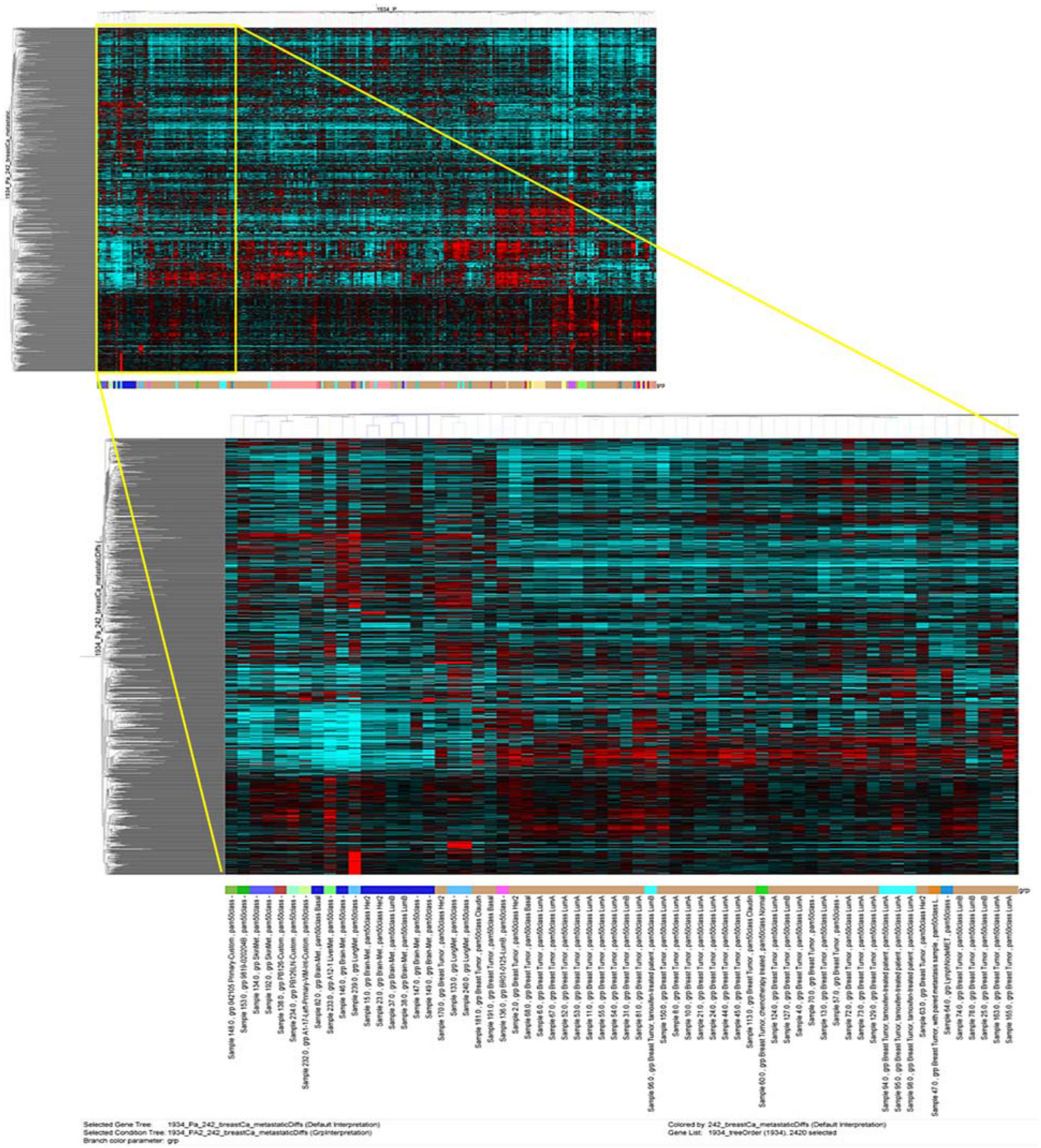

Supplementary Figure 4.2: Gene expression in primary tumor and various metastatic sites. Analysis of the GSE26338 data set. Heat map of gene expression in primary breast cancer versus various metastatic sites. The metastases cluster to the left. The upper panel shows all specimens, while the lower panel zooms in on the area that clusters primary tumors close to metastatic tumors. A downregulation in the expression of individual genes is reflected in bright blue in the heat map.

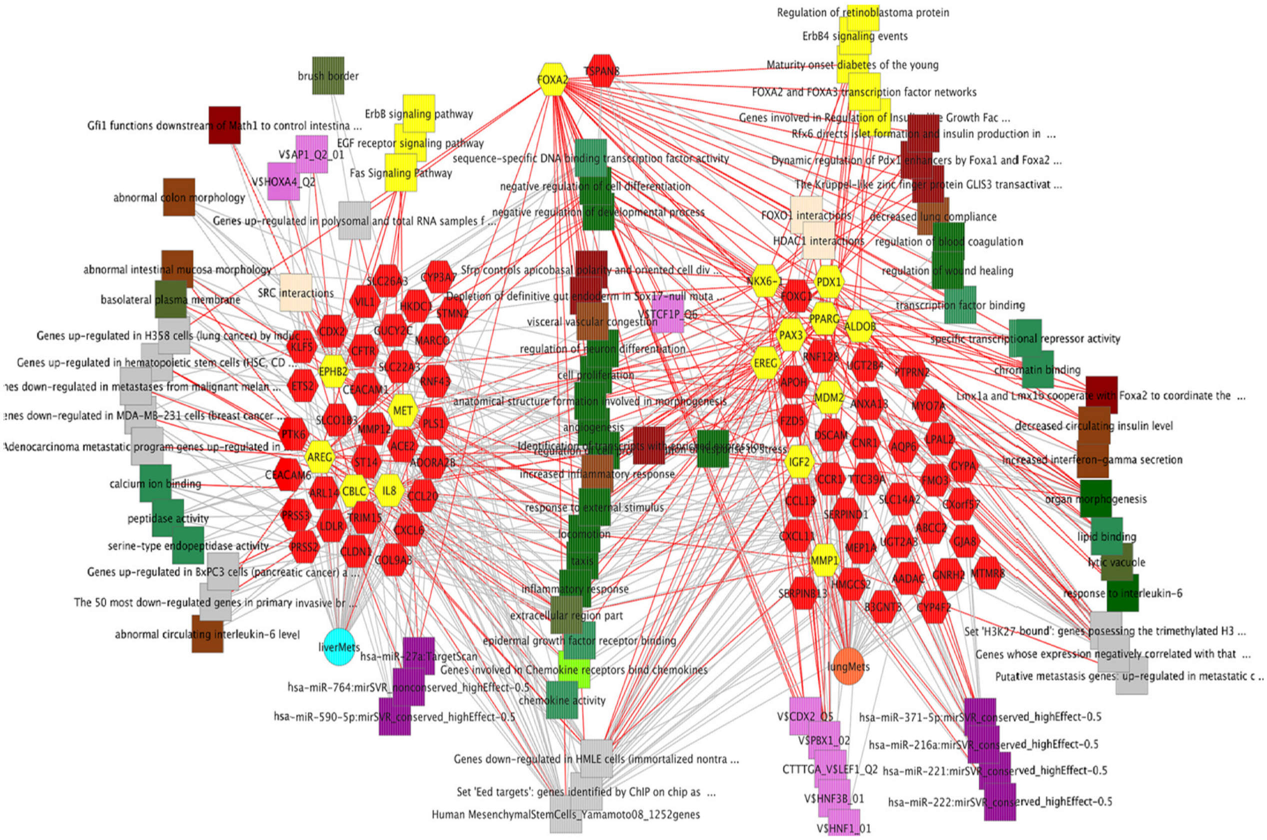

Supplementary Figure 5: Pathway analysis. Principal pathways and functions that are specific for or shared between the two different signature gene lists of colorectal cancer metastases to the liver (left) or to the lungs (right) respectively. The pathways are selected at the top (yellow squares), and the genes that they are connected to are yellow hexagons. These genes are connected to all the other significant features and functions by red edges. The gray edges of those other functions are all the other genes of the lists that are also very connected to those pathways.
